# The synthesis, crystal structure and Hirshfeld surface analysis of the thio­phene derivatives 5-(phenyl­sulfon­yl)-5,6-di­hydro­benzo[4,5]thieno[3,2-*j*]phenanthridine and (*E*)-*N*-{2-[2-(benzo[*b*]thiophen-2-yl)ethenyl]phen­yl}-*N*-(prop-2-yn-1-yl)benzene­sulfonamide

**DOI:** 10.1107/S2056989023003821

**Published:** 2023-05-05

**Authors:** S. Madhan, M. NizamMohideen, Vinayagam Pavunkumar, Arasambattu K. MohanaKrishnan

**Affiliations:** a Department of Physics, The New College, Chennai 600 014, University of Madras, Tamil Nadu, India; b Department of Organic Chemistry, University of Madras, Guindy Campus, Chennai-600 025, Tamilnadu, India; Venezuelan Institute of Scientific Research, Venezuela

**Keywords:** crystal structure, thio­phene, benzo­thio­phene, di­hydro­benzene, di­hydro­pyridine 5-(phenyl­sulfon­yl), 12-(phenyl­sulfon­yl), phenanthridine hydrogen bonding, Hirshfeld surface analysis.

## Abstract

In both of the title compounds, C_26_H_19_NO_2_S_2_, (**I**), and C_25_H_19_NO_2_S_2_, (**II**), the benzo­thio­phene rings are essentially planar with maximum deviations of 0.026 (1) and −0.016 (1) Å for the carbon and sulfur atoms in compounds (**I**) and (**II**), respectively. In (**I**), the thio­phene ring system is almost orthogonal to the phenyl ring attached to the sulfonyl group, subtending a dihedral angle of 88.1 (1)°, and the di­hydro­pyridine ring adopts a screw–boat conformation. In both compounds, the mol­ecular structure is consolidated by weak C—H⋯O intra­molecular inter­actions formed by the sulfone oxygen atoms, which generate *S*(5) ring motifs. In the crystal of **II**, mol­ecules are linked *via* C—H⋯O hydrogen bonds, generating *C*(7) chains running along the [100] direction. No significant inter­molecular inter­actions are observed in **I**.

## Abstract 2

The crystal structures of two benzo­thio­phene derivatives are described along with an analysis of the inter­molecular contacts in the crystals performed using Hirshfeld surface analysis and two-dimensional fingerprint plots.

## 1. Chemical context

Thio­phene, C_4_H_4_S, belongs to a class of aromatic five-membered heterocycles comprising one S heteroatom. Thio­phene derivatives possess pharmacological and biological activities including anti­bacterial (Mishra *et al.*, 2012 ▸), anti­allergic (Gillespie *et al.*,1985 ▸), anti-cancer and anti-toxic (Gewald *et al.*, 1966 ▸), analgesic (Laddi *et al.*, 1998 ▸; Chen *et al.*, 2008 ▸), anti-inflammatory (Ferreira *et al.*, 2006 ▸), anti­oxidant (Jarak *et al.*, 2005 ▸), anti­tumor (Gadad *et al.*, 1994 ▸), anti­microbial (Abdel-Rahman *et al.*, 2003 ▸), anti­hypertensive (Monge Vega *et al.*, 1980 ▸), anti-diabetes mellitus (Abdelhamid *et al.*, 2009 ▸), gonadotropin releasing hormone antagonist (Sabins *et al.*, 1944 ▸) and are building blocks in many agrochemicals (Ansary & Omar, 2001 ▸). Thio­phene possesses promising pharmacological activities, such as anti-HIV PR inhibitor (Bonini *et al.*, 2005 ▸) and anti-breast cancer (Brault *et al.*, 2005 ▸). Benzo­thio­phenes are biologically energetic mol­ecules. One of the most significant drugs based on the benzo­thio­phene structure is Raloxifine, used for the stoppage and cure of osteoporosis in postmenopausal women (Jordan, 2003 ▸). Benzo­thio­phenes are also present in luminescent components used in organic materials (Russell & Press, 1996 ▸). Thio­phene derivatives have a wide variety of applications in optical and electronic systems (Gather *et al.*, 2008 ▸; He *et al.*, 2009 ▸) and are used extensively in solar cells (Justin Thomas *et al.*, 2008 ▸), organic light-emitting diodes (OLEDs) (Mazzeo *et al.*, 2003 ▸), organic field-effect transistors (OFETs) (Zhan *et al.*, 2007 ▸) and as NLO devices (Bedworth *et al.*, 1996 ▸; Raposo *et al.*, 2011 ▸). Thieno-pyridine products are used in medicine as allosteric adenosine receptors and in the treatment of adenosine-sensitive cardiac arrhythmias (Tumey *et al.*, 2008 ▸; Grunewald *et al.*, 2008 ▸). Herein we report the crystal structure and Hirshfeld surface analysis of the title thio­phene derivatives. 
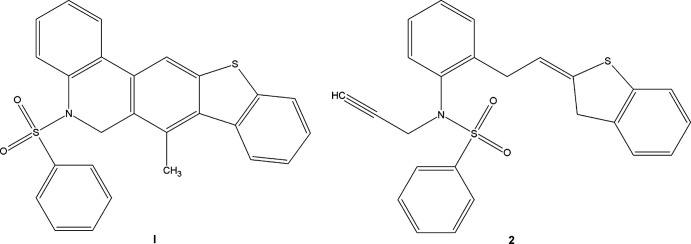


## 2. Structural commentary

**Figure 1:**
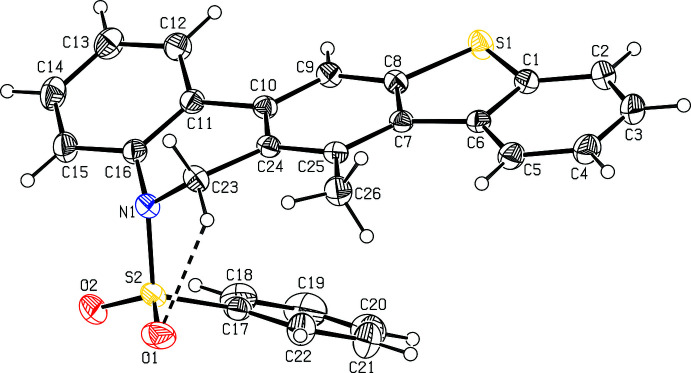
The mol­ecular structure of compound **I**, with atom labelling. Displacement ellipsoids are drawn at the 30% probability level. Intra­molecular contacts are shown as dashed lines (Table 1 ▸).

**Figure 2:**
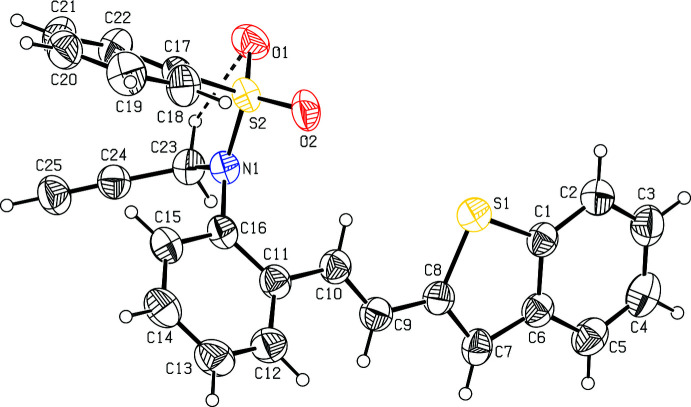
The mol­ecular structure of compound **II**, with atom labelling. Displacement ellipsoids are drawn at the 30% probability level. Intra­molecular contacts are shown as dashed lines (Table 2 ▸).

The mol­ecular structure of compound **I** (Fig. 1 ▸) comprises a benzo­thio­phene ring system (S1/C1–C8) attached to a 4-methyl 5-(phenyl­sulfon­yl)-5,6-di­hydro­phenanthridine unit (C7–C26/N1/S2/O1/O2), while compound **II** comprises a benzo­thio­phene ring system (S1/C1–C8) attached to an *N*-(2-allyl­phen­yl)-*N*-prop-2-yn-1-yl benzene­sulfonamide group (C9–C15/N1/S2/O1/O2) (Fig. 2 ▸). In both compounds, the benzo­thio­phene ring system (S1/C1–C8) is essentially planar with maximum deviations of 0.026 (1) and 0.016 (1) Å for atom C6 and S1 in compounds **I** and **II**, respectively. The mean planes of the thio­phene ring systems make dihedral angles of 2.1 (1), 19.0 (1) and 33.9 (1), respectively, in compound **I** and 0.7 (2), 38.1 (2) and 87.6 (2)°, respectively, in compound **II** with the C1–C6, C11–C16 and C17–C22 phenyl rings. The benzo­thio­phene ring system is almost orthogonal to the C17–C22 phenyl ring attached to the sulfonyl group in **I**, subtending a dihedral angle of 88.1 (1)°, while the di­hydro­pyridine ring (C10/C11/C16/C23/C24) adopts a screw–boat conformation, as is evident from the Cremer–Pople puckering analysis of the six-membered heterocyclic ring [*Q* = 0.4451 (13) Å, θ = 111.5 (2) and φ = 146.9 (2)°]

In both compounds, the tetra­hedral configuration is distorted around the atom S2. The increase in the O2—S2—O1 angle [120.0 (1)° in **I** and 119.9 (2)° in **II**], with a simultaneous decrease in the N1—S2—C17 angle [108.5 (1)° in **I** and 107.6 (1)° in **II**] from the ideal tetra­hedral value (109.5°) are attributed to the Thorpe–Ingold effect (Bassindale, 1984 ▸). The widening of the angles may be due to the repulsive inter­action between the two short S=O bonds. The N1—C23 [1.477 (2) Å in **I** and 1.477 (3) Å in **II**] and N1—C16 [1.433 (2) Å in **I** and 1.444 (3) Å in (**II**] bond lengths in the mol­ecule are longer than the mean N*sp* ^2^—C*sp* ^2^ bond length value of 1.355 (14) Å (Allen *et al.*, 1987 ▸; Cambridge Structural Database (CSD) Version 5.37; Groom *et al.*, 2016 ▸). The elongation observed may be due to the electron-withdrawing character of the phenyl­sulfonyl group. The sum of the bond angles around N1 [350.2° in **I** and 357.6° in **II**]] indicate the *sp* ^2^ hybridization. The geometric parameters of compounds **I** and **II** agree well with those reported for related structures (Madhan *et al.*, 2022 ▸).

In both compounds, the mol­ecular structure is stabilized by weak C23—H23⋯O1 intra­molecular inter­actions (Tables 1 ▸ and 2 ▸) formed by the sulfone oxygen atoms, which generate *S*(5) ring motifs (Figs. 1 ▸ and 2 ▸).

## 3. Supra­molecular features

In the crystal of **I**, weak π–π inter­actions are present [*Cg*1⋯*Cg*2^i^ = 3.766 (2) Å where *Cg*1 and *Cg*2 are the centroids of rings S1/C1/C6–C8 and C7–C10/C24/C25, respectively; symmetry code: (i) 1 - **x**, 1 - **y**, 1 - **z**]. No significant inter­molecular inter­actions or C—H⋯π inter­actions with centroid distances of less than 4 Å are observed in the structure.

**Figure 3:**
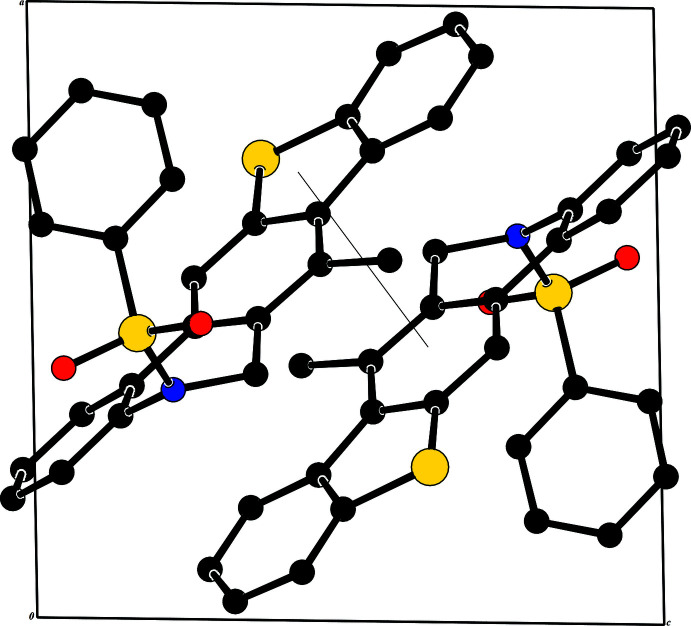
A view along the *b-*axis of the crystal packing of compound **I**. The hydrogen bonds are shown as dashed lines (Table 1 ▸), and H atoms not involved in hydrogen bonding have been omitted.

In the crystal of **II**, mol­ecules are linked *via* C25—H25⋯O1 hydrogen bonding, generating *C*(7) chains (Bernstein *et al.*, 1995 ▸) running along the [100] direction. Weak π–π [*Cg*3⋯*Cg*3^ii^ = 3.649 (2) Å where *Cg*3 is the centroid of the S1/C1/C6–C8 ring; symmetry code: (ii) −*x*, 2 − *y*, 1 − *z*] and C—H⋯π inter­actions [C21—H21⋯*Cg*4^iii^ where *Cg*4 is the centroid of the C1—C6 ring; symmetry code: (iii) 1 − *x*, 1 − *y*, 1 − *z*]. are also present. Packing view of the title compound are shown in Figs. 3 ▸ and 4 ▸.

## 4. Hirshfeld surface analysis

A recent article by Tiekink and collaborators (Tan *et al.*, 2019 ▸) reviews and describes the uses and utility of Hirshfeld surface analysis (Spackman & Jayatilaka, 2009 ▸) and the associated two-dimensional fingerprint plots (McKinnon *et al.*, 2007 ▸) to analyse inter­molecular contacts in crystals. The various calculations (*d*_norm_, curvedness and shape index and 2D fingerprint plots) were performed with *CrystalExplorer17* (Turner *et al.*, 2017 ▸).

**Figure 5:**
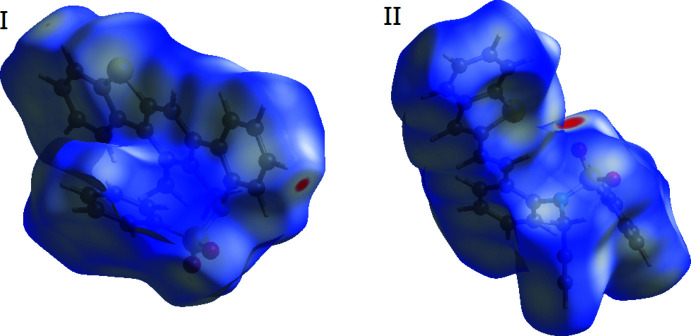
The Hirshfeld surfaces of compounds **I** and **II**, mapped over *d*_norm_

The Hirshfeld surfaces of compounds **I** and **II** mapped over *d*_norm_ are shown in Fig. 5 ▸. They are colour-mapped with the normalized contact distance, *d*_norm_, from red (distances shorter than the sum of the van der Waals radii) through white to blue (distances longer than the sum of the van der Waals radii). The *d*_norm_ surface was mapped over a fixed colour scale of −0.085 (red) to 1.564 (blue) for compound **I** and −0.286 (red) to 1.374 (blue) for compound **II**. The red spots indicate inter­molecular contacts involved in hydrogen bonding.

**Figure 6:**
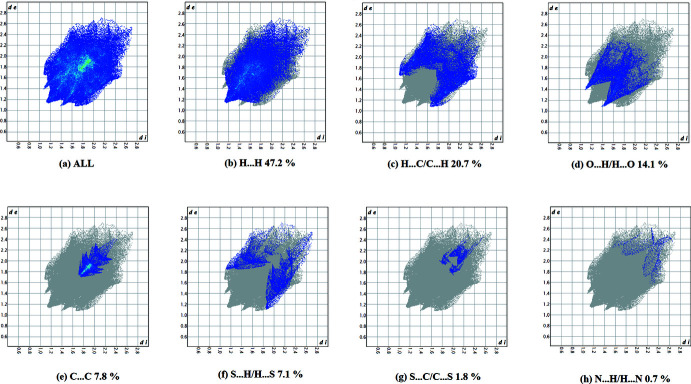
The full two-dimensional fingerprint plot for compound **I**, and fingerprint plots delineated into (*b*) H⋯H, (*c*) O⋯H/H⋯O, (*d*) C⋯H/H⋯C, (*e*) C⋯C and (*f*) N⋯H/H⋯N contacts.

**Figure 7:**
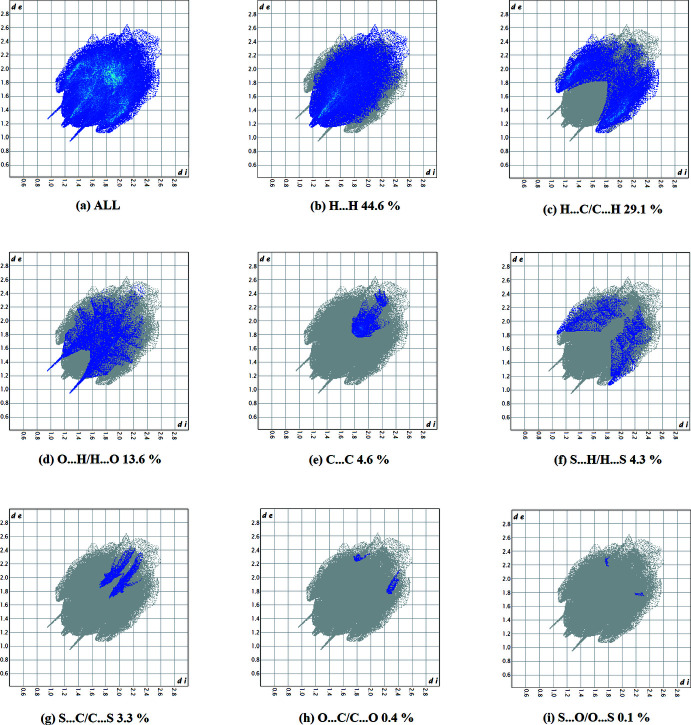
The full two-dimensional fingerprint plot for compound **II**, and fingerprint plots delineated into (*b*) C⋯C, (*c*) C⋯H/H⋯C, (*d*) C⋯·N/N⋯C, (*e*) C⋯O/O⋯C, (*f*) H⋯H, (*g*) N⋯H/H⋯N, (*h*) O⋯H/H⋯O and (I) S⋯H/H⋯S contacts.

The fingerprint plots are illustrated in Figs. 6 ▸ and 7 ▸. For compound **I**, they reveal that the principal inter­molecular contacts are H⋯H (47.2%, Fig. 6 ▸ *b*), H⋯C/C⋯H (20.7%, Fig. 6 ▸ *c*), O⋯H/H⋯O (14.1%, Fig. 6 ▸ *d*), C⋯C (7.8%, Fig. 6 ▸ *e*), S⋯H/H⋯S (7.4%, Fig. 6 ▸ *f*), S⋯C/C⋯S (1.8%, Fig. 6 ▸ *g*) and N⋯H/H⋯N (0.7%, Fig. 6 ▸ *h*). For compound **II**, they reveal a similar trend, with the principal inter­molecular contacts being H⋯H/H⋯H (44.6%, Fig. 7 ▸ *b*), H⋯C/C⋯H (29.1%, Fig. 7 ▸ *c*), O⋯H/H⋯O (13.6%, Fig. 7 ▸ *d*), C⋯C (4.6%, Fig. 7 ▸ *e*), S⋯H/H⋯S (4.3%, Fig. 7 ▸ *f*), S⋯C/C⋯S (3.3%, Fig. 7 ▸ *g*), C⋯O/O⋯C (0.4%, Fig. 7 ▸ *h*) and S⋯O/O⋯S (0.1%, Fig. 7 ▸ *i*). In both compounds, the H⋯H inter­molecular contacts predominate, followed by C⋯H/H⋯C and O⋯H/H⋯O contacts.

## 5. Synthesis and crystallization

Compound **I**: A solution of *N*-propargyl­benzene­sulfonamide (0.50 g) in xylenes (20 mL), MnO_2_ (0.50 g) was added and the reaction mixture was refluxed for 24 h. It was then filtered through a celite pad and washed with hot xylenes (2 × 10 mL). The combined filtrate was concentrated under vacuum and then triturated with MeOH to afford dibenzo[*b*]thio­phene (0.38 g, 92%) as a dull white solid. Finally, compound **I** was crystallized using ethanol.

Compound **II**: To a solution of (*E*)-*N*-{2-[2-(benzo[*b*]thio­phen-2-yl)ethenyl]phen­yl}benzenesulfonamide (1.2 g, 3.069 mmol) in CH_3_CN (10 mL), K_2_CO_3_ (0.63 g, 4.603 mmol) and propargyl bromide (0.54 mL, 4.603 mmol) were added and the mixture was stirred at room temperature for 12 h. After completion of the reaction (monitored by TLC), it was poured into crushed ice (50 g) containing conc. HCl (5 mL), extracted with ethyl acetate (2 × 20 mL) then washed with water (2 × 20 mL) and dried (Na_2_SO_4_). Removal of the solvent *in vacuo* followed by crystallization from methanol (4 mL) afforded compound **II** as a white solid.

## 6. Refinement

Crystal data, data collection and structure refinement details are summarized in Table 3 ▸. H atoms were found difference electron-density maps and positioned geometrically. They were refined as riding, with C—H = 0.93–0.94 Å and *U*iso(H) = 1.2*U*eq(C) or 1.5*U*eq(Cmeth­yl).

## Supplementary Material

Crystal structure: contains datablock(s) global, I, II. DOI: 10.1107/S2056989023003821/zn2028sup1.cif

Structure factors: contains datablock(s) I. DOI: 10.1107/S2056989023003821/zn2028Isup2.hkl

Structure factors: contains datablock(s) II. DOI: 10.1107/S2056989023003821/zn2028IIsup3.hkl

Supporting information file. DOI: 10.1107/S2056989023003821/zn2028Isup4.cml

Supporting information file. DOI: 10.1107/S2056989023003821/zn2028IIsup5.cml

CCDC references: 2259716, 2259715

Additional supporting information:  crystallographic information; 3D view; checkCIF report

## Figures and Tables

**Figure 4:**
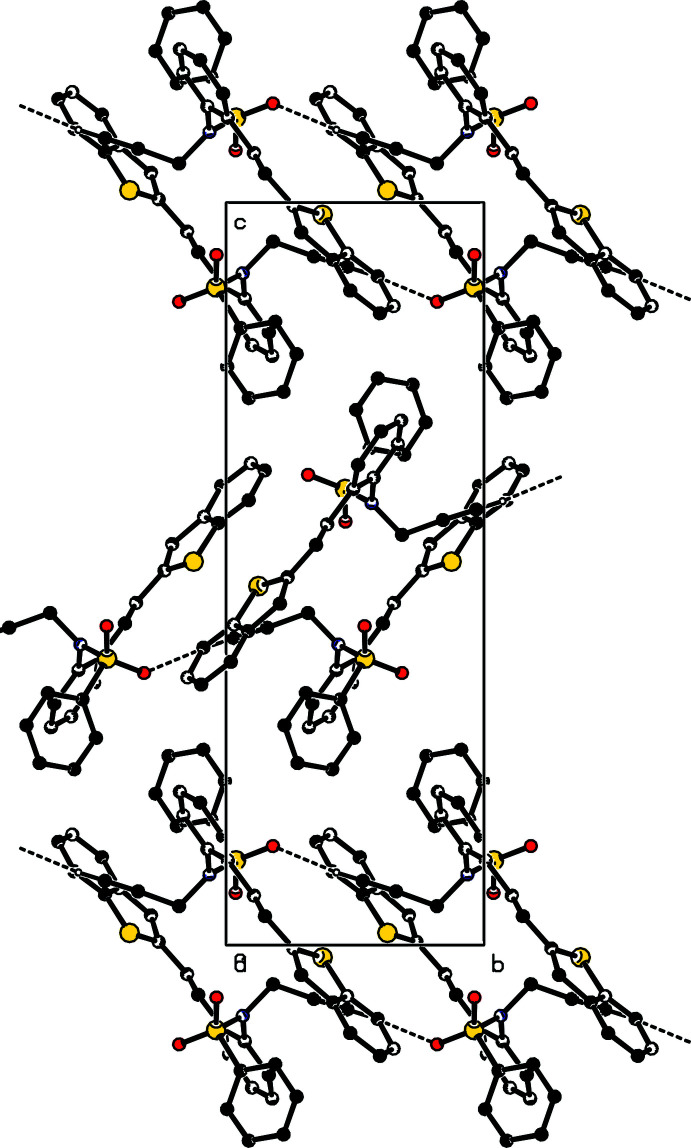
A view along the *a-*axis of the crystal packing of compound **II**. The hydrogen bonds are shown as dashed lines (Table 2 ▸), and H atoms not involved in hydrogen bonding have been omitted.

**Table 1:** Hydrogen-bond geometry (Å, °) for **I** [Chem scheme1]

*D*—H⋯*A*	*D*—H	H⋯*A*	*D*⋯*A*	*D*—H⋯*A*
C23—H23*A*⋯O1	0.97	2.41	2.8935 (17)	111

**Table 2:** Hydrogen-bond geometry (Å, °) for **II** [Chem scheme1] *Cg*1 is the centroid of the C1–C6 ring.

*D*—H⋯*A*	*D*—H	H⋯*A*	*D*⋯*A*	*D*—H⋯*A*
C23—H23*A*⋯O1	0.97	2.35	2.860 (3)	112
C25—H25⋯O2^i^	0.93	2.38	3.285 (4)	166
C21—H21⋯*Cg*1^ii^	0.93	2.97	3.772 (2)	139

Symmetry codes: (i) 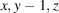 ; (ii) 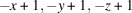 .

**Table 3:** Experimental details

	**I**	**II**
Crystal data		
Chemical formula	C_26_H_19_NO_2_S_2_	C_25_H_19_NO_2_S_2_
*M*_r_	441.54	429.53
Crystal system, space group	Monoclinic, *P*2_1_/*n*	Monoclinic, *P*2_1_/*c*
Temperature (K)	298	298
*a*, *b*, *c* (Å)	10.3378 (4), 19.4299 (7), 10.5128 (4)	10.0085 (4), 8.6474 (3), 24.9024 (10)
β (°)	91.666 (1)	91.560 (2)
*V* (Å^3^)	2110.73 (14)	2154.44 (14)
*Z*	4	4
Radiation type	Mo *K*α	Cu *K*α
μ (mm^−1^)	0.28	2.41
Crystal size (mm)	0.28 × 0.21 × 0.11	0.55 × 0.16 × 0.09

Data collection		
Diffractometer	Bruker D8 VENTURE with PHOTON II detector	Bruker D8 VENTURE with PHOTON II detector
Absorption correction	Multi-scan (*SADABS*; Krause *et al.*, 2015 ▸)	Multi-scan (*SADABS*; Krause *et al.*, 2015 ▸)
*T*_min_, *T*_max_	0.913, 0.952	0.473, 0.752
No. of measured, independent and observed [*I* > 2σ(*I*)] reflections	102594, 6426, 5503	51958, 4099, 3475
*R*_int_	0.049	0.062
(sin θ/λ)_max_ (Å^−1^)	0.714	0.610

Refinement		
*R*[*F* ^2^ > 2σ(*F* ^2^)], *wR*(*F* ^2^), *S*	0.039, 0.107, 1.08	0.053, 0.176, 1.08
No. of reflections	6426	4099
No. of parameters	281	272
No. of restraints	0	79
H-atom treatment	H-atom parameters constrained	H-atom parameters constrained
Δρ_max_, Δρ_min_ (e Å^−3^)	0.28, −0.41	0.48, −0.44

Computer programs: *APEX3*, *SAINT* and *XPREP* (Bruker, 2016 ▸), *SHELXT2018/2* (Sheldrick, 2015*a* ▸), *SHELXL2018/3* (Sheldrick, 2015*b* ▸), *ORTEP-3 for Windows* and *WinGX* (Farrugia, 2012 ▸), *Mercury* (Macrae *et al.*, 2020 ▸), *publCIF* (Westrip, 2010 ▸) and *PLATON* (Spek, 2020 ▸).

## supplementary crystallographic information

### 5-(Phenylsulfonyl)-5,6-dihydrobenzo[4,5]thieno[3,2-j]phenanthridine (I) Crystal data

**Table:**

C_26_H_19_NO_2_S_2_	*F*(000) = 920
*M**_r_* = 441.54	*D*_x_ = 1.389 Mg m^−^^3^
Monoclinic, *P*2_1_/*n*	Mo *K*α radiation, λ = 0.71073 Å
*a* = 10.3378 (4) Å	Cell parameters from 9927 reflections
*b* = 19.4299 (7) Å	θ = 2.7–30.5°
*c* = 10.5128 (4) Å	µ = 0.28 mm^−^^1^
β = 91.666 (1)°	*T* = 298 K
*V* = 2110.73 (14) Å^3^	Solid, white
*Z* = 4	0.28 × 0.21 × 0.11 mm

### 5-(Phenylsulfonyl)-5,6-dihydrobenzo[4,5]thieno[3,2-j]phenanthridine (I) Data collection

**Table:**

Bruker D8 VENTURE diffractometer with PHOTON II detector	6426 independent reflections
Radiation source: fine-focus sealed tube	5503 reflections with *I* > 2σ(*I*)
Graphite monochromator	*R*_int_ = 0.049
ω and φ scan	θ_max_ = 30.5°, θ_min_ = 3.4°
Absorption correction: multi-scan (SADABS; Krause *et al.*, 2015)	*h* = −14→14
*T*_min_ = 0.913, *T*_max_ = 0.952	*k* = −27→27
102594 measured reflections	*l* = −15→15

### 5-(Phenylsulfonyl)-5,6-dihydrobenzo[4,5]thieno[3,2-j]phenanthridine (I) Refinement

**Table:**

Refinement on *F*^2^	0 restraints
Least-squares matrix: full	Hydrogen site location: inferred from neighbouring sites
*R*[*F*^2^ > 2σ(*F*^2^)] = 0.039	H-atom parameters constrained
*wR*(*F*^2^) = 0.107	*w* = 1/[σ^2^(*F*_o_^2^) + (0.0461*P*)^2^ + 0.647*P*] where *P* = (*F*_o_^2^ + 2*F*_c_^2^)/3
*S* = 1.08	(Δ/σ)_max_ = 0.001
6426 reflections	Δρ_max_ = 0.28 e Å^−^^3^
281 parameters	Δρ_min_ = −0.41 e Å^−^^3^

### 5-(Phenylsulfonyl)-5,6-dihydrobenzo[4,5]thieno[3,2-j]phenanthridine (I) Special details

**Table:**

Geometry. All esds (except the esd in the dihedral angle between two l.s. planes) are estimated using the full covariance matrix. The cell esds are taken into account individually in the estimation of esds in distances, angles and torsion angles; correlations between esds in cell parameters are only used when they are defined by crystal symmetry. An approximate (isotropic) treatment of cell esds is used for estimating esds involving l.s. planes.

### 5-(Phenylsulfonyl)-5,6-dihydrobenzo[4,5]thieno[3,2-j]phenanthridine (I) Fractional atomic coordinates and isotropic or equivalent isotropic displacement parameters (Å^2^)

**Table:**

	*x*	*y*	*z*	*U*_iso_*/*U*_eq_	
C1	0.18150 (12)	0.54394 (7)	0.49074 (12)	0.0357 (2)	
C2	0.07837 (14)	0.57488 (8)	0.42365 (15)	0.0456 (3)	
H2	0.043585	0.616286	0.450922	0.055*	
C3	0.02956 (15)	0.54248 (9)	0.31610 (16)	0.0509 (4)	
H3	−0.038513	0.562400	0.269510	0.061*	
C4	0.08113 (15)	0.48034 (9)	0.27666 (15)	0.0486 (3)	
H4	0.046942	0.459128	0.203845	0.058*	
C5	0.18211 (13)	0.44951 (7)	0.34353 (13)	0.0411 (3)	
H5	0.214467	0.407556	0.316150	0.049*	
C6	0.23642 (11)	0.48106 (6)	0.45270 (11)	0.0323 (2)	
C7	0.34011 (11)	0.45888 (6)	0.54103 (11)	0.0304 (2)	
C8	0.35613 (12)	0.50602 (6)	0.64208 (12)	0.0326 (2)	
C9	0.44637 (12)	0.49656 (6)	0.74072 (12)	0.0345 (2)	
H9	0.454519	0.528641	0.806122	0.041*	
C10	0.52436 (11)	0.43836 (6)	0.74018 (11)	0.0314 (2)	
C11	0.62210 (12)	0.42551 (6)	0.84265 (11)	0.0335 (2)	
C12	0.66959 (14)	0.47761 (8)	0.92326 (13)	0.0421 (3)	
H12	0.639586	0.522406	0.912326	0.051*	
C13	0.76051 (15)	0.46350 (9)	1.01902 (14)	0.0484 (3)	
H13	0.790176	0.498700	1.072230	0.058*	
C14	0.80733 (15)	0.39763 (9)	1.03599 (15)	0.0504 (3)	
H14	0.868110	0.388423	1.100704	0.060*	
C15	0.76402 (14)	0.34548 (8)	0.95703 (14)	0.0460 (3)	
H15	0.796669	0.301174	0.967394	0.055*	
C16	0.67120 (12)	0.35912 (7)	0.86155 (12)	0.0356 (2)	
C17	0.38346 (13)	0.27810 (7)	0.86487 (13)	0.0395 (3)	
C18	0.36218 (19)	0.30659 (9)	0.98291 (17)	0.0586 (4)	
H18	0.427937	0.307864	1.045138	0.070*	
C19	0.2403 (2)	0.33334 (11)	1.0068 (2)	0.0768 (6)	
H19	0.224407	0.352937	1.085500	0.092*	
C20	0.1442 (2)	0.33097 (11)	0.9155 (3)	0.0786 (7)	
H20	0.062980	0.348623	0.932799	0.094*	
C21	0.16574 (18)	0.30305 (12)	0.7993 (2)	0.0745 (6)	
H21	0.099453	0.302077	0.737596	0.089*	
C22	0.28611 (16)	0.27605 (9)	0.77249 (17)	0.0548 (4)	
H22	0.300957	0.256810	0.693241	0.066*	
C23	0.60124 (13)	0.32934 (7)	0.64517 (12)	0.0373 (3)	
H23A	0.562993	0.291924	0.595934	0.045*	
H23B	0.682220	0.341678	0.606776	0.045*	
C24	0.51108 (11)	0.39059 (6)	0.63939 (11)	0.0308 (2)	
C25	0.42157 (11)	0.40052 (6)	0.53871 (11)	0.0310 (2)	
C26	0.41359 (15)	0.35107 (7)	0.42818 (14)	0.0442 (3)	
H26A	0.483215	0.318491	0.435605	0.066*	
H26B	0.420076	0.376139	0.349937	0.066*	
H26C	0.332397	0.327105	0.428648	0.066*	
N1	0.62789 (10)	0.30555 (5)	0.77677 (11)	0.0366 (2)	
O1	0.52000 (12)	0.19506 (5)	0.73109 (12)	0.0559 (3)	
O2	0.59493 (12)	0.22243 (6)	0.95103 (12)	0.0598 (3)	
S1	0.25152 (3)	0.57621 (2)	0.63062 (3)	0.04119 (9)	
S2	0.53669 (3)	0.24354 (2)	0.83244 (3)	0.03996 (9)	

### 5-(Phenylsulfonyl)-5,6-dihydrobenzo[4,5]thieno[3,2-j]phenanthridine (I) Atomic displacement parameters (Å^2^)

**Table:**

	*U*^11^	*U*^22^	*U*^33^	*U*^12^	*U*^13^	*U*^23^
C1	0.0340 (6)	0.0350 (6)	0.0382 (6)	−0.0006 (5)	0.0008 (5)	0.0048 (5)
C2	0.0410 (7)	0.0425 (7)	0.0529 (8)	0.0062 (6)	−0.0040 (6)	0.0076 (6)
C3	0.0417 (7)	0.0546 (9)	0.0556 (9)	0.0016 (6)	−0.0110 (6)	0.0124 (7)
C4	0.0447 (7)	0.0553 (8)	0.0450 (7)	−0.0054 (6)	−0.0110 (6)	0.0019 (6)
C5	0.0399 (6)	0.0429 (7)	0.0401 (6)	−0.0030 (5)	−0.0039 (5)	−0.0014 (5)
C6	0.0304 (5)	0.0324 (5)	0.0341 (5)	−0.0035 (4)	0.0018 (4)	0.0042 (4)
C7	0.0312 (5)	0.0291 (5)	0.0308 (5)	−0.0025 (4)	0.0022 (4)	0.0026 (4)
C8	0.0345 (5)	0.0291 (5)	0.0341 (6)	0.0018 (4)	0.0016 (4)	0.0003 (4)
C9	0.0392 (6)	0.0324 (5)	0.0317 (5)	0.0008 (5)	−0.0001 (5)	−0.0019 (4)
C10	0.0335 (5)	0.0310 (5)	0.0299 (5)	−0.0013 (4)	0.0016 (4)	0.0027 (4)
C11	0.0328 (5)	0.0360 (6)	0.0318 (5)	−0.0019 (4)	0.0010 (4)	0.0033 (4)
C12	0.0459 (7)	0.0422 (7)	0.0380 (6)	−0.0018 (6)	−0.0037 (5)	−0.0010 (5)
C13	0.0462 (7)	0.0593 (9)	0.0392 (7)	−0.0046 (6)	−0.0062 (6)	−0.0048 (6)
C14	0.0412 (7)	0.0665 (10)	0.0428 (7)	0.0025 (7)	−0.0098 (6)	0.0050 (7)
C15	0.0396 (7)	0.0506 (8)	0.0475 (7)	0.0050 (6)	−0.0056 (6)	0.0087 (6)
C16	0.0313 (5)	0.0381 (6)	0.0375 (6)	−0.0012 (5)	0.0013 (4)	0.0065 (5)
C17	0.0397 (6)	0.0320 (6)	0.0473 (7)	−0.0015 (5)	0.0079 (5)	0.0045 (5)
C18	0.0632 (10)	0.0598 (10)	0.0535 (9)	−0.0070 (8)	0.0155 (8)	−0.0043 (7)
C19	0.0836 (14)	0.0679 (12)	0.0814 (14)	0.0007 (10)	0.0436 (12)	−0.0087 (10)
C20	0.0536 (10)	0.0634 (11)	0.1207 (19)	0.0110 (9)	0.0367 (12)	0.0130 (12)
C21	0.0432 (9)	0.0744 (13)	0.1058 (17)	0.0080 (9)	−0.0021 (10)	0.0128 (12)
C22	0.0463 (8)	0.0574 (9)	0.0607 (9)	0.0047 (7)	−0.0002 (7)	0.0022 (8)
C23	0.0416 (6)	0.0341 (6)	0.0363 (6)	0.0052 (5)	0.0036 (5)	0.0023 (5)
C24	0.0329 (5)	0.0276 (5)	0.0319 (5)	−0.0006 (4)	0.0029 (4)	0.0025 (4)
C25	0.0325 (5)	0.0278 (5)	0.0327 (5)	−0.0025 (4)	0.0023 (4)	0.0000 (4)
C26	0.0489 (7)	0.0383 (7)	0.0449 (7)	0.0040 (6)	−0.0072 (6)	−0.0102 (5)
N1	0.0359 (5)	0.0325 (5)	0.0412 (6)	0.0013 (4)	0.0004 (4)	0.0062 (4)
O1	0.0604 (7)	0.0323 (5)	0.0755 (8)	−0.0006 (5)	0.0138 (6)	−0.0093 (5)
O2	0.0557 (6)	0.0548 (6)	0.0683 (7)	0.0017 (5)	−0.0060 (5)	0.0319 (6)
S1	0.04479 (18)	0.03577 (16)	0.04275 (18)	0.00946 (13)	−0.00302 (13)	−0.00405 (12)
S2	0.03959 (17)	0.02892 (14)	0.05148 (19)	0.00299 (11)	0.00343 (13)	0.00800 (12)

### 5-(Phenylsulfonyl)-5,6-dihydrobenzo[4,5]thieno[3,2-j]phenanthridine (I) Geometric parameters (Å, º)

**Table:**

C1—C2	1.3970 (18)	C15—C16	1.3937 (18)
C1—C6	1.4100 (18)	C15—H15	0.9300
C1—S1	1.7372 (14)	C16—N1	1.4334 (17)
C2—C3	1.377 (2)	C17—C22	1.379 (2)
C2—H2	0.9300	C17—C18	1.382 (2)
C3—C4	1.388 (2)	C17—S2	1.7629 (14)
C3—H3	0.9300	C18—C19	1.393 (3)
C4—C5	1.378 (2)	C18—H18	0.9300
C4—H4	0.9300	C19—C20	1.361 (3)
C5—C6	1.4038 (17)	C19—H19	0.9300
C5—H5	0.9300	C20—C21	1.361 (3)
C6—C7	1.4625 (16)	C20—H20	0.9300
C7—C8	1.4088 (16)	C21—C22	1.387 (2)
C7—C25	1.4132 (16)	C21—H21	0.9300
C8—C9	1.3868 (17)	C22—H22	0.9300
C8—S1	1.7426 (12)	C23—N1	1.4770 (16)
C9—C10	1.3890 (17)	C23—C24	1.5117 (16)
C9—H9	0.9300	C23—H23A	0.9700
C10—C24	1.4123 (16)	C23—H23B	0.9700
C10—C11	1.4767 (17)	C24—C25	1.3988 (16)
C11—C16	1.3984 (17)	C25—C26	1.5081 (17)
C11—C12	1.3998 (18)	C26—H26A	0.9600
C12—C13	1.385 (2)	C26—H26B	0.9600
C12—H12	0.9300	C26—H26C	0.9600
C13—C14	1.378 (2)	N1—S2	1.6478 (11)
C13—H13	0.9300	O1—S2	1.4289 (12)
C14—C15	1.376 (2)	O2—S2	1.4288 (11)
C14—H14	0.9300		
			
C2—C1—C6	122.51 (13)	C11—C16—N1	118.42 (11)
C2—C1—S1	124.41 (11)	C22—C17—C18	120.79 (15)
C6—C1—S1	113.05 (9)	C22—C17—S2	119.63 (12)
C3—C2—C1	118.29 (14)	C18—C17—S2	119.57 (13)
C3—C2—H2	120.9	C17—C18—C19	118.66 (19)
C1—C2—H2	120.9	C17—C18—H18	120.7
C2—C3—C4	120.54 (13)	C19—C18—H18	120.7
C2—C3—H3	119.7	C20—C19—C18	120.39 (19)
C4—C3—H3	119.7	C20—C19—H19	119.8
C5—C4—C3	121.15 (14)	C18—C19—H19	119.8
C5—C4—H4	119.4	C21—C20—C19	120.74 (18)
C3—C4—H4	119.4	C21—C20—H20	119.6
C4—C5—C6	120.41 (14)	C19—C20—H20	119.6
C4—C5—H5	119.8	C20—C21—C22	120.3 (2)
C6—C5—H5	119.8	C20—C21—H21	119.9
C5—C6—C1	117.09 (12)	C22—C21—H21	119.9
C5—C6—C7	131.20 (12)	C17—C22—C21	119.13 (18)
C1—C6—C7	111.65 (11)	C17—C22—H22	120.4
C8—C7—C25	118.53 (10)	C21—C22—H22	120.4
C8—C7—C6	110.80 (10)	N1—C23—C24	112.46 (10)
C25—C7—C6	130.66 (11)	N1—C23—H23A	109.1
C9—C8—C7	122.65 (11)	C24—C23—H23A	109.1
C9—C8—S1	123.92 (9)	N1—C23—H23B	109.1
C7—C8—S1	113.43 (9)	C24—C23—H23B	109.1
C8—C9—C10	118.87 (11)	H23A—C23—H23B	107.8
C8—C9—H9	120.6	C25—C24—C10	121.66 (11)
C10—C9—H9	120.6	C25—C24—C23	122.17 (11)
C9—C10—C24	119.63 (11)	C10—C24—C23	116.16 (10)
C9—C10—C11	121.31 (11)	C24—C25—C7	118.62 (10)
C24—C10—C11	119.06 (11)	C24—C25—C26	121.02 (11)
C16—C11—C12	117.50 (12)	C7—C25—C26	120.34 (11)
C16—C11—C10	119.83 (11)	C25—C26—H26A	109.5
C12—C11—C10	122.67 (11)	C25—C26—H26B	109.5
C13—C12—C11	121.04 (14)	H26A—C26—H26B	109.5
C13—C12—H12	119.5	C25—C26—H26C	109.5
C11—C12—H12	119.5	H26A—C26—H26C	109.5
C14—C13—C12	120.43 (14)	H26B—C26—H26C	109.5
C14—C13—H13	119.8	C16—N1—C23	113.68 (10)
C12—C13—H13	119.8	C16—N1—S2	118.79 (9)
C15—C14—C13	119.92 (13)	C23—N1—S2	117.93 (9)
C15—C14—H14	120.0	C1—S1—C8	91.06 (6)
C13—C14—H14	120.0	O2—S2—O1	120.00 (8)
C14—C15—C16	119.97 (14)	O2—S2—N1	106.79 (6)
C14—C15—H15	120.0	O1—S2—N1	105.97 (7)
C16—C15—H15	120.0	O2—S2—C17	107.39 (7)
C15—C16—C11	121.13 (13)	O1—S2—C17	107.80 (7)
C15—C16—N1	120.39 (12)	N1—S2—C17	108.47 (6)
			
C6—C1—C2—C3	−0.3 (2)	C18—C19—C20—C21	−0.6 (3)
S1—C1—C2—C3	−177.95 (11)	C19—C20—C21—C22	0.5 (3)
C1—C2—C3—C4	0.6 (2)	C18—C17—C22—C21	0.0 (3)
C2—C3—C4—C5	−0.1 (2)	S2—C17—C22—C21	179.65 (14)
C3—C4—C5—C6	−0.9 (2)	C20—C21—C22—C17	−0.2 (3)
C4—C5—C6—C1	1.19 (19)	C9—C10—C24—C25	0.50 (17)
C4—C5—C6—C7	178.32 (13)	C11—C10—C24—C25	−179.03 (11)
C2—C1—C6—C5	−0.62 (19)	C9—C10—C24—C23	179.03 (11)
S1—C1—C6—C5	177.28 (10)	C11—C10—C24—C23	−0.50 (16)
C2—C1—C6—C7	−178.29 (12)	N1—C23—C24—C25	−146.17 (11)
S1—C1—C6—C7	−0.39 (13)	N1—C23—C24—C10	35.31 (15)
C5—C6—C7—C8	−176.35 (13)	C10—C24—C25—C7	−2.03 (17)
C1—C6—C7—C8	0.90 (14)	C23—C24—C25—C7	179.53 (11)
C5—C6—C7—C25	3.9 (2)	C10—C24—C25—C26	176.61 (12)
C1—C6—C7—C25	−178.82 (12)	C23—C24—C25—C26	−1.83 (18)
C25—C7—C8—C9	−1.59 (18)	C8—C7—C25—C24	2.51 (16)
C6—C7—C8—C9	178.66 (11)	C6—C7—C25—C24	−177.79 (11)
C25—C7—C8—S1	178.72 (9)	C8—C7—C25—C26	−176.14 (11)
C6—C7—C8—S1	−1.03 (13)	C6—C7—C25—C26	3.56 (19)
C7—C8—C9—C10	0.05 (19)	C15—C16—N1—C23	−143.71 (12)
S1—C8—C9—C10	179.71 (9)	C11—C16—N1—C23	33.72 (16)
C8—C9—C10—C24	0.51 (18)	C15—C16—N1—S2	70.73 (15)
C8—C9—C10—C11	−179.97 (11)	C11—C16—N1—S2	−111.84 (11)
C9—C10—C11—C16	160.90 (12)	C24—C23—N1—C16	−52.19 (14)
C24—C10—C11—C16	−19.59 (17)	C24—C23—N1—S2	93.69 (12)
C9—C10—C11—C12	−19.40 (19)	C2—C1—S1—C8	177.69 (12)
C24—C10—C11—C12	160.12 (12)	C6—C1—S1—C8	−0.16 (10)
C16—C11—C12—C13	−0.8 (2)	C9—C8—S1—C1	−178.99 (11)
C10—C11—C12—C13	179.48 (13)	C7—C8—S1—C1	0.70 (10)
C11—C12—C13—C14	0.7 (2)	C16—N1—S2—O2	−45.38 (11)
C12—C13—C14—C15	0.3 (2)	C23—N1—S2—O2	170.52 (10)
C13—C14—C15—C16	−1.2 (2)	C16—N1—S2—O1	−174.40 (10)
C14—C15—C16—C11	1.1 (2)	C23—N1—S2—O1	41.49 (11)
C14—C15—C16—N1	178.41 (13)	C16—N1—S2—C17	70.08 (11)
C12—C11—C16—C15	−0.07 (19)	C23—N1—S2—C17	−74.03 (11)
C10—C11—C16—C15	179.65 (12)	C22—C17—S2—O2	−152.99 (13)
C12—C11—C16—N1	−177.47 (11)	C18—C17—S2—O2	26.65 (14)
C10—C11—C16—N1	2.25 (17)	C22—C17—S2—O1	−22.39 (14)
C22—C17—C18—C19	−0.1 (3)	C18—C17—S2—O1	157.25 (12)
S2—C17—C18—C19	−179.73 (14)	C22—C17—S2—N1	91.93 (13)
C17—C18—C19—C20	0.4 (3)	C18—C17—S2—N1	−88.43 (13)

### 5-(Phenylsulfonyl)-5,6-dihydrobenzo[4,5]thieno[3,2-j]phenanthridine (I) Hydrogen-bond geometry (Å, º)

**Table:**

*D*—H···*A*	*D*—H	H···*A*	*D*···*A*	*D*—H···*A*
C23—H23*A*···O1	0.97	2.41	2.8935 (17)	111

### (E)-N-{2-[2-(Benzo[b]thiophen-2-yl)ethenyl]phenyl}-\ N-(prop-2-yn-1-yl)benzenesulfonamide (II) Crystal data

**Table:**

C_25_H_19_NO_2_S_2_	*F*(000) = 896
*M**_r_* = 429.53	*D*_x_ = 1.324 Mg m^−^^3^
Monoclinic, *P*2_1_/*c*	Cu *K*α radiation, λ = 1.54178 Å
*a* = 10.0085 (4) Å	Cell parameters from 9879 reflections
*b* = 8.6474 (3) Å	θ = 3.6–70.0°
*c* = 24.9024 (10) Å	µ = 2.41 mm^−^^1^
β = 91.560 (2)°	*T* = 298 K
*V* = 2154.44 (14) Å^3^	Solid, white
*Z* = 4	0.55 × 0.16 × 0.09 mm

### (E)-N-{2-[2-(Benzo[b]thiophen-2-yl)ethenyl]phenyl}-\ N-(prop-2-yn-1-yl)benzenesulfonamide (II) Data collection

**Table:**

Bruker D8 VENTURE diffractometer with PHOTON II detector	4099 independent reflections
Radiation source: micro-focus sealed tube	3475 reflections with *I* > 2σ(*I*)
Graphite monochromator	*R*_int_ = 0.062
ω and φ scan	θ_max_ = 70.2°, θ_min_ = 3.6°
Absorption correction: multi-scan (SADABS; Krause *et al.*, 2015)	*h* = −12→12
*T*_min_ = 0.473, *T*_max_ = 0.752	*k* = −10→9
51958 measured reflections	*l* = −30→30

### (E)-N-{2-[2-(Benzo[b]thiophen-2-yl)ethenyl]phenyl}-\ N-(prop-2-yn-1-yl)benzenesulfonamide (II) Refinement

**Table:**

Refinement on *F*^2^	Hydrogen site location: inferred from neighbouring sites
Least-squares matrix: full	H-atom parameters constrained
*R*[*F*^2^ > 2σ(*F*^2^)] = 0.053	*w* = 1/[σ^2^(*F*_o_^2^) + (0.1029*P*)^2^ + 0.5764*P*] where *P* = (*F*_o_^2^ + 2*F*_c_^2^)/3
*wR*(*F*^2^) = 0.176	(Δ/σ)_max_ = 0.001
*S* = 1.08	Δρ_max_ = 0.48 e Å^−^^3^
4099 reflections	Δρ_min_ = −0.44 e Å^−^^3^
272 parameters	Extinction correction: SHELXL2018/3 (Sheldrick 2015b), Fc^*^=kFc[1+0.001xFc^2^λ^3^/sin(2θ)]^-1/4^
79 restraints	Extinction coefficient: 0.0022 (5)

### (E)-N-{2-[2-(Benzo[b]thiophen-2-yl)ethenyl]phenyl}-\ N-(prop-2-yn-1-yl)benzenesulfonamide (II) Special details

**Table:**

Geometry. All esds (except the esd in the dihedral angle between two l.s. planes) are estimated using the full covariance matrix. The cell esds are taken into account individually in the estimation of esds in distances, angles and torsion angles; correlations between esds in cell parameters are only used when they are defined by crystal symmetry. An approximate (isotropic) treatment of cell esds is used for estimating esds involving l.s. planes.

### (E)-N-{2-[2-(Benzo[b]thiophen-2-yl)ethenyl]phenyl}-\ N-(prop-2-yn-1-yl)benzenesulfonamide (II) Fractional atomic coordinates and isotropic or equivalent isotropic displacement parameters (Å^2^)

**Table:**

	*x*	*y*	*z*	*U*_iso_*/*U*_eq_	
C1	0.1532 (3)	0.9693 (3)	0.56842 (11)	0.0684 (6)	
C2	0.2089 (3)	1.0879 (3)	0.59929 (13)	0.0840 (7)	
H2	0.294440	1.124310	0.592867	0.101*	
C3	0.1350 (4)	1.1498 (4)	0.63931 (13)	0.0920 (9)	
H3	0.170944	1.229010	0.660409	0.110*	
C4	0.0074 (4)	1.0964 (4)	0.64899 (13)	0.0936 (9)	
H4	−0.040772	1.139698	0.676637	0.112*	
C5	−0.0482 (3)	0.9815 (4)	0.61851 (12)	0.0855 (8)	
H5	−0.134220	0.947089	0.625262	0.103*	
C6	0.0238 (3)	0.9148 (3)	0.57697 (10)	0.0682 (6)	
C7	−0.0194 (3)	0.7911 (3)	0.54011 (10)	0.0716 (6)	
H7	−0.101598	0.741005	0.539835	0.086*	
C8	0.0883 (2)	0.7615 (3)	0.50449 (10)	0.0668 (5)	
C9	0.0850 (3)	0.6508 (3)	0.46095 (11)	0.0714 (6)	
H9	0.003002	0.606209	0.451630	0.086*	
C10	0.1907 (3)	0.6081 (3)	0.43325 (11)	0.0702 (6)	
H10	0.273528	0.645705	0.444964	0.084*	
C11	0.1883 (3)	0.5069 (3)	0.38578 (10)	0.0674 (6)	
C12	0.0736 (3)	0.4882 (4)	0.35331 (12)	0.0824 (7)	
H12	−0.004815	0.537783	0.362753	0.099*	
C13	0.0734 (4)	0.3992 (4)	0.30808 (13)	0.0926 (9)	
H13	−0.004121	0.390672	0.286857	0.111*	
C14	0.1880 (3)	0.3213 (4)	0.29349 (12)	0.0899 (8)	
H14	0.187589	0.261028	0.262562	0.108*	
C15	0.3024 (3)	0.3341 (3)	0.32527 (11)	0.0745 (6)	
H15	0.378979	0.280031	0.316357	0.089*	
C16	0.3032 (3)	0.4275 (3)	0.37049 (9)	0.0642 (5)	
C17	0.6191 (2)	0.4432 (3)	0.33097 (10)	0.0648 (6)	
C18	0.5938 (3)	0.4956 (4)	0.27912 (12)	0.0847 (8)	
H18	0.545850	0.586357	0.273075	0.102*	
C19	0.6404 (4)	0.4114 (4)	0.23677 (13)	0.0974 (9)	
H19	0.624473	0.446264	0.201853	0.117*	
C20	0.7100 (3)	0.2772 (4)	0.24517 (14)	0.0903 (8)	
H20	0.739550	0.220487	0.216061	0.108*	
C21	0.7358 (3)	0.2265 (4)	0.29602 (14)	0.0920 (9)	
H21	0.783481	0.135327	0.301581	0.110*	
C22	0.6919 (3)	0.3095 (3)	0.33956 (12)	0.0793 (7)	
H22	0.711203	0.275587	0.374343	0.095*	
C23	0.4365 (3)	0.3176 (3)	0.44743 (10)	0.0693 (6)	
H23A	0.518561	0.337577	0.467869	0.083*	
H23B	0.362892	0.327825	0.471735	0.083*	
C24	0.4395 (3)	0.1591 (3)	0.42754 (11)	0.0708 (6)	
C25	0.4400 (3)	0.0303 (3)	0.41334 (13)	0.0878 (8)	
H25	0.440438	−0.072212	0.402039	0.105*	
N1	0.4216 (2)	0.4356 (2)	0.40484 (8)	0.0625 (5)	
O1	0.6444 (2)	0.5370 (2)	0.42964 (8)	0.0864 (6)	
O2	0.4958 (2)	0.6818 (2)	0.36649 (9)	0.0859 (6)	
S1	0.22906 (7)	0.87417 (8)	0.51653 (3)	0.0780 (3)	
S2	0.54870 (6)	0.53848 (6)	0.38576 (3)	0.0679 (2)	

### (E)-N-{2-[2-(Benzo[b]thiophen-2-yl)ethenyl]phenyl}-\ N-(prop-2-yn-1-yl)benzenesulfonamide (II) Atomic displacement parameters (Å^2^)

**Table:**

	*U*^11^	*U*^22^	*U*^33^	*U*^12^	*U*^13^	*U*^23^
C1	0.0725 (14)	0.0600 (13)	0.0728 (14)	0.0080 (10)	0.0014 (11)	0.0061 (10)
C2	0.0822 (17)	0.0739 (16)	0.0956 (19)	0.0037 (13)	−0.0014 (14)	−0.0053 (14)
C3	0.104 (2)	0.0820 (19)	0.090 (2)	0.0154 (16)	−0.0054 (16)	−0.0158 (15)
C4	0.113 (2)	0.090 (2)	0.0791 (18)	0.0294 (18)	0.0150 (16)	0.0007 (15)
C5	0.0834 (18)	0.0852 (19)	0.0888 (19)	0.0133 (14)	0.0187 (15)	0.0150 (14)
C6	0.0710 (13)	0.0624 (13)	0.0713 (13)	0.0079 (10)	0.0056 (11)	0.0115 (9)
C7	0.0822 (15)	0.0609 (13)	0.0713 (14)	0.0129 (11)	−0.0028 (10)	0.0017 (10)
C8	0.0695 (13)	0.0599 (13)	0.0707 (13)	0.0046 (10)	−0.0010 (10)	0.0069 (10)
C9	0.0721 (14)	0.0657 (14)	0.0761 (15)	0.0010 (11)	−0.0025 (11)	0.0040 (11)
C10	0.0737 (14)	0.0618 (13)	0.0747 (15)	0.0051 (11)	−0.0055 (11)	0.0006 (11)
C11	0.0758 (14)	0.0581 (12)	0.0679 (14)	−0.0008 (11)	−0.0039 (11)	0.0035 (10)
C12	0.0791 (17)	0.0826 (18)	0.0847 (18)	0.0014 (14)	−0.0116 (14)	0.0015 (14)
C13	0.094 (2)	0.098 (2)	0.0850 (19)	−0.0117 (17)	−0.0227 (16)	−0.0017 (16)
C14	0.108 (2)	0.0894 (19)	0.0717 (16)	−0.0138 (17)	−0.0103 (15)	−0.0140 (14)
C15	0.0880 (16)	0.0663 (14)	0.0691 (14)	−0.0072 (12)	−0.0006 (12)	−0.0074 (11)
C16	0.0757 (14)	0.0539 (11)	0.0626 (12)	−0.0039 (10)	−0.0037 (10)	0.0008 (9)
C17	0.0708 (13)	0.0551 (12)	0.0687 (13)	−0.0066 (10)	0.0020 (10)	0.0013 (10)
C18	0.109 (2)	0.0689 (15)	0.0761 (16)	0.0056 (15)	0.0015 (15)	0.0091 (13)
C19	0.123 (3)	0.102 (2)	0.0677 (17)	−0.003 (2)	0.0072 (16)	−0.0004 (15)
C20	0.0948 (19)	0.086 (2)	0.0911 (19)	−0.0089 (16)	0.0239 (16)	−0.0161 (16)
C21	0.094 (2)	0.0775 (18)	0.105 (2)	0.0143 (15)	0.0232 (17)	−0.0014 (15)
C22	0.0834 (17)	0.0761 (16)	0.0788 (16)	0.0148 (13)	0.0079 (13)	0.0071 (13)
C23	0.0798 (15)	0.0680 (14)	0.0601 (13)	0.0038 (11)	0.0001 (11)	0.0001 (10)
C24	0.0745 (14)	0.0642 (14)	0.0737 (15)	−0.0011 (11)	0.0010 (11)	0.0082 (11)
C25	0.102 (2)	0.0614 (16)	0.099 (2)	−0.0030 (14)	−0.0069 (17)	0.0033 (14)
N1	0.0703 (11)	0.0545 (10)	0.0625 (11)	−0.0011 (8)	−0.0004 (8)	−0.0017 (8)
O1	0.0862 (12)	0.0927 (14)	0.0794 (12)	−0.0152 (10)	−0.0116 (9)	−0.0167 (10)
O2	0.1087 (14)	0.0464 (9)	0.1030 (14)	0.0004 (9)	0.0092 (11)	−0.0023 (9)
S1	0.0729 (4)	0.0769 (5)	0.0842 (5)	0.0002 (3)	0.0065 (3)	−0.0032 (3)
S2	0.0782 (4)	0.0514 (3)	0.0740 (4)	−0.0057 (2)	0.0004 (3)	−0.0089 (2)

### (E)-N-{2-[2-(Benzo[b]thiophen-2-yl)ethenyl]phenyl}-\ N-(prop-2-yn-1-yl)benzenesulfonamide (II) Geometric parameters (Å, º)

**Table:**

C1—C2	1.389 (4)	C14—C15	1.379 (4)
C1—C6	1.400 (4)	C14—H14	0.9300
C1—S1	1.725 (3)	C15—C16	1.386 (3)
C2—C3	1.366 (5)	C15—H15	0.9300
C2—H2	0.9300	C16—N1	1.444 (3)
C3—C4	1.385 (5)	C17—C22	1.380 (4)
C3—H3	0.9300	C17—C18	1.385 (4)
C4—C5	1.361 (5)	C17—S2	1.758 (3)
C4—H4	0.9300	C18—C19	1.374 (5)
C5—C6	1.401 (4)	C18—H18	0.9300
C5—H5	0.9300	C19—C20	1.366 (5)
C6—C7	1.467 (4)	C19—H19	0.9300
C7—C8	1.437 (4)	C20—C21	1.358 (5)
C7—H7	0.9300	C20—H20	0.9300
C8—C9	1.446 (4)	C21—C22	1.382 (4)
C8—S1	1.733 (3)	C21—H21	0.9300
C9—C10	1.331 (4)	C22—H22	0.9300
C9—H9	0.9300	C23—C24	1.457 (4)
C10—C11	1.471 (4)	C23—N1	1.477 (3)
C10—H10	0.9300	C23—H23A	0.9700
C11—C12	1.396 (4)	C23—H23B	0.9700
C11—C16	1.401 (4)	C24—C25	1.169 (4)
C12—C13	1.364 (5)	C25—H25	0.9300
C12—H12	0.9300	N1—S2	1.634 (2)
C13—C14	1.386 (5)	O1—S2	1.433 (2)
C13—H13	0.9300	O2—S2	1.426 (2)
			
C2—C1—C6	121.6 (3)	C14—C15—C16	120.0 (3)
C2—C1—S1	126.0 (2)	C14—C15—H15	120.0
C6—C1—S1	112.4 (2)	C16—C15—H15	120.0
C3—C2—C1	118.4 (3)	C15—C16—C11	121.3 (2)
C3—C2—H2	120.8	C15—C16—N1	119.8 (2)
C1—C2—H2	120.8	C11—C16—N1	118.8 (2)
C2—C3—C4	121.1 (3)	C22—C17—C18	120.1 (3)
C2—C3—H3	119.4	C22—C17—S2	119.6 (2)
C4—C3—H3	119.4	C18—C17—S2	120.2 (2)
C5—C4—C3	120.8 (3)	C19—C18—C17	119.0 (3)
C5—C4—H4	119.6	C19—C18—H18	120.5
C3—C4—H4	119.6	C17—C18—H18	120.5
C4—C5—C6	120.1 (3)	C20—C19—C18	121.0 (3)
C4—C5—H5	120.0	C20—C19—H19	119.5
C6—C5—H5	120.0	C18—C19—H19	119.5
C1—C6—C5	118.1 (3)	C21—C20—C19	120.0 (3)
C1—C6—C7	114.1 (2)	C21—C20—H20	120.0
C5—C6—C7	127.8 (3)	C19—C20—H20	120.0
C8—C7—C6	107.7 (2)	C20—C21—C22	120.5 (3)
C8—C7—H7	126.2	C20—C21—H21	119.8
C6—C7—H7	126.2	C22—C21—H21	119.8
C7—C8—C9	125.3 (2)	C17—C22—C21	119.4 (3)
C7—C8—S1	114.4 (2)	C17—C22—H22	120.3
C9—C8—S1	120.28 (19)	C21—C22—H22	120.3
C10—C9—C8	124.7 (3)	C24—C23—N1	114.1 (2)
C10—C9—H9	117.6	C24—C23—H23A	108.7
C8—C9—H9	117.6	N1—C23—H23A	108.7
C9—C10—C11	125.9 (3)	C24—C23—H23B	108.7
C9—C10—H10	117.1	N1—C23—H23B	108.7
C11—C10—H10	117.1	H23A—C23—H23B	107.6
C12—C11—C16	117.0 (2)	C25—C24—C23	177.5 (3)
C12—C11—C10	122.0 (2)	C24—C25—H25	180.0
C16—C11—C10	121.0 (2)	C16—N1—C23	117.24 (19)
C13—C12—C11	121.7 (3)	C16—N1—S2	119.12 (16)
C13—C12—H12	119.1	C23—N1—S2	121.40 (17)
C11—C12—H12	119.1	C1—S1—C8	91.47 (13)
C12—C13—C14	120.5 (3)	O2—S2—O1	119.89 (13)
C12—C13—H13	119.7	O2—S2—N1	106.65 (11)
C14—C13—H13	119.7	O1—S2—N1	106.44 (12)
C15—C14—C13	119.4 (3)	O2—S2—C17	107.41 (12)
C15—C14—H14	120.3	O1—S2—C17	108.30 (13)
C13—C14—H14	120.3	N1—S2—C17	107.60 (11)
			
C6—C1—C2—C3	1.0 (4)	C22—C17—C18—C19	0.9 (5)
S1—C1—C2—C3	−179.4 (2)	S2—C17—C18—C19	−174.4 (3)
C1—C2—C3—C4	−0.2 (5)	C17—C18—C19—C20	0.6 (5)
C2—C3—C4—C5	−0.5 (5)	C18—C19—C20—C21	−1.1 (5)
C3—C4—C5—C6	0.4 (5)	C19—C20—C21—C22	0.2 (5)
C2—C1—C6—C5	−1.1 (4)	C18—C17—C22—C21	−1.8 (4)
S1—C1—C6—C5	179.3 (2)	S2—C17—C22—C21	173.6 (2)
C2—C1—C6—C7	179.0 (2)	C20—C21—C22—C17	1.2 (5)
S1—C1—C6—C7	−0.6 (3)	C15—C16—N1—C23	−86.4 (3)
C4—C5—C6—C1	0.4 (4)	C11—C16—N1—C23	89.3 (3)
C4—C5—C6—C7	−179.8 (3)	C15—C16—N1—S2	76.8 (3)
C1—C6—C7—C8	−0.1 (3)	C11—C16—N1—S2	−107.4 (2)
C5—C6—C7—C8	−180.0 (2)	C24—C23—N1—C16	59.7 (3)
C6—C7—C8—C9	−178.1 (2)	C24—C23—N1—S2	−103.1 (2)
C6—C7—C8—S1	0.8 (3)	C2—C1—S1—C8	−178.7 (3)
C7—C8—C9—C10	−169.8 (3)	C6—C1—S1—C8	0.87 (19)
S1—C8—C9—C10	11.4 (4)	C7—C8—S1—C1	−1.0 (2)
C8—C9—C10—C11	−174.1 (2)	C9—C8—S1—C1	178.0 (2)
C9—C10—C11—C12	23.3 (4)	C16—N1—S2—O2	45.4 (2)
C9—C10—C11—C16	−158.3 (3)	C23—N1—S2—O2	−152.10 (18)
C16—C11—C12—C13	−1.2 (4)	C16—N1—S2—O1	174.46 (17)
C10—C11—C12—C13	177.2 (3)	C23—N1—S2—O1	−23.0 (2)
C11—C12—C13—C14	1.3 (5)	C16—N1—S2—C17	−69.6 (2)
C12—C13—C14—C15	0.2 (5)	C23—N1—S2—C17	92.91 (19)
C13—C14—C15—C16	−1.8 (5)	C22—C17—S2—O2	172.2 (2)
C14—C15—C16—C11	1.9 (4)	C18—C17—S2—O2	−12.4 (3)
C14—C15—C16—N1	177.5 (2)	C22—C17—S2—O1	41.4 (2)
C12—C11—C16—C15	−0.4 (4)	C18—C17—S2—O1	−143.2 (2)
C10—C11—C16—C15	−178.8 (2)	C22—C17—S2—N1	−73.3 (2)
C12—C11—C16—N1	−176.0 (2)	C18—C17—S2—N1	102.1 (2)
C10—C11—C16—N1	5.5 (3)		

### (E)-N-{2-[2-(Benzo[b]thiophen-2-yl)ethenyl]phenyl}-\ N-(prop-2-yn-1-yl)benzenesulfonamide (II) Hydrogen-bond geometry (Å, º)

Cg1 is the centroid of the C1–C6 ring.

**Table:**

*D*—H···*A*	*D*—H	H···*A*	*D*···*A*	*D*—H···*A*
C23—H23*A*···O1	0.97	2.35	2.860 (3)	112
C25—H25···O2^i^	0.93	2.38	3.285 (4)	166
C21—H21···*Cg*1^ii^	0.93	2.97	3.772 (2)	139

Symmetry codes: (i) *x*, *y*−1, *z*; (ii) −*x*+1, −*y*+1, −*z*+1.
